# Exclusively Digital Health Interventions Targeting Diet, Physical Activity, and Weight Gain in Pregnant Women: Systematic Review and Meta-Analysis

**DOI:** 10.2196/18255

**Published:** 2020-07-10

**Authors:** Alexandra Rhodes, Andrea D Smith, Paul Chadwick, Helen Croker, Clare H Llewellyn

**Affiliations:** 1 University College London London United Kingdom

**Keywords:** gestational weight gain, digital interventions, behavior change techniques, user engagement, smartphone, mobile phone

## Abstract

**Background:**

Interventions to promote a healthy diet, physical activity, and weight management during pregnancy are increasingly embracing digital technologies. Although some interventions have combined digital with interpersonal (face-to-face or telephone) delivery, others have relied exclusively on digital delivery. Exclusively digital interventions have the advantages of greater cost-effectiveness and broader reach and as such can be a valuable resource for health care providers.

**Objective:**

This systematic review aims to focus on exclusively digital interventions to determine their effectiveness, identify behavior change techniques (BCTs), and investigate user engagement.

**Methods:**

A total of 6 databases (Medical Literature Analysis and Retrieval System Online [MEDLINE], Excerpta Medica dataBASE [EMBASE], PsycINFO, Cumulated Index to Nursing and Allied Health Literature [CINAHL] Plus, Web of Science, and ProQuest) were searched for randomized controlled trials or pilot control trials of exclusively digital interventions to encourage healthy eating, physical activity, or appropriate weight gain during pregnancy. The outcome measures were gestational weight gain (GWG) and changes in physical activity and dietary behaviors. Study quality was assessed using the Cochrane Risk of Bias tool 2.0. Where possible, pooled effect sizes were calculated using a random effects meta-analysis.

**Results:**

In total, 11 studies met the inclusion criteria. The risk of bias was mostly high (n=5) or moderate (n=3). Of the 11 studies, 6 reported on GWG as the primary outcome, 4 of which also measured changes in physical activity and dietary behaviors, and 5 studies focused either on dietary behaviors only (n=2) or physical activity only (n=3). The meta-analyses showed no significant benefit of interventions on total GWG for either intention-to-treat data (−0.28 kg; 95% CI −1.43 to 0.87) or per-protocol data (−0.65 kg; 95% CI −1.98 to 0.67). Substantial heterogeneity in outcome measures of change in dietary behaviors and physical activity precluded further meta-analyses. BCT coding identified 7 BCTs that were common to all effective interventions. Effective interventions averaged over twice as many BCTs from the *goals and planning*, and *feedback and monitoring* domains as ineffective interventions. Data from the 6 studies reporting on user engagement indicated a positive association between high engagement with key BCTs and greater intervention effectiveness. Interventions using proactive messaging and feedback appeared to have higher levels of engagement.

**Conclusions:**

In contrast to interpersonal interventions, there is little evidence of the effectiveness of exclusively digital interventions to encourage a healthy diet, physical activity, or weight management during pregnancy. In this review, effective interventions used proactive messaging, such as reminders to engage in BCTs, feedback on progress, or tips, suggesting that interactivity may drive engagement and lead to greater effectiveness. Given the benefits of cost and reach of digital interventions, further research is needed to understand how to use advancing technologies to enhance user engagement and improve effectiveness.

## Introduction

### Background

Poor diet and lack of physical activity are 21st century problems contributing to the obesity crisis. In the United Kingdom, more than 50% of women of childbearing age are estimated to have overweight or obesity [1]. Pregnancy has been identified as a teachable moment [2], when women may be motivated to make lifestyle changes to improve their own health and the health of their unborn baby. Encouraging women to improve their diet and levels of physical activity is beneficial for not only supporting healthy gestational weight gain (GWG) and maternal health [3] but also developing behaviors that may potentially improve the health of the whole family.

In many countries, GWG is monitored, and women are given guidance for recommended levels of weight gain [4]. The most widely used GWG guidelines are the US Institute of Medicine (IOM) 2009 guidelines, where the recommended range of weight gain is based on a woman’s pre-pregnancy BMI [5]. It is estimated that in many high-income countries, more than 50% of women gain excessive weight during pregnancy [6,7]. This is problematic because excessive GWG is associated with an increased risk of adverse health outcomes, such as gestational diabetes, large for gestational age babies, macrosomia, and cesarean section [6,8]. GWG is also associated with an increased risk of postpartum weight retention, which increases the likelihood of starting subsequent pregnancies with overweight or obesity [9]. The effects of excessive GWG are also believed to have an intergenerational impact, increasing the likelihood of overweight and obesity throughout the life of the baby [10,11]. In the United Kingdom, most women are not routinely weighed during pregnancy nor are they given specific advice on healthy levels of weight gain. However, they are provided with general advice to eat a healthy diet and participate in regular physical activity [12].

Interventions targeting diet, physical activity, or both to encourage healthier lifestyles during pregnancy and reduce rates of excessive GWG have been shown to be effective, with diet-only interventions leading to greater weight reductions than physical activity alone or combined interventions [13-15]. A recent review of systematic reviews and meta-analyses reported reductions in GWG between 0.7 and 1.8 kg, as well as positive effects on maternal and infant health outcomes [16]. The majority of these lifestyle interventions used interpersonal delivery, either in person or telephone. Several more recent interventions have embraced digital delivery methods, recognizing their advantages of significantly lower costs and broader reach [17]. A self-managed digital intervention that can be delivered anytime and anywhere and at a lower cost to the patient and provider can be a valuable resource for health care providers, provided it can affect positive behavior change.

In nonpregnant populations, digital interventions have been shown to be effective in changing nutritional behaviors [18], encouraging weight management [19], and improving levels of physical activity [20]. However, evidence of their effectiveness in improving lifestyle behaviors during pregnancy is mixed. In the past 3 years, 5 systematic reviews have explored various aspects of digital interventions to improve diet, increase physical activity, or manage weight during pregnancy [14,17,21-23]. Of the 4 including meta-analyses, 2 found no significant effect of the interventions [14,21], whereas one showed a significant result for limiting GWG, increasing physical activity, and reducing dietary energy intake in women with overweight or obesity [17] and another found a moderate effect on managing GWG among women of all BMIs [23]. However, except for Lau et al [17], who conducted a subgroup meta-analysis comprising 2 studies, these systematic reviews did not distinguish between interventions that combined digital with an interpersonal element of coaching or support (the majority of studies) and those that were exclusively digital. Moreover, although one review reported on usability [22], systematic reviews to date have not investigated user engagement, a vital component of self-managed digital interventions [24]. Finally, although previous systematic reviews have explored the behavior change techniques (BCTs) [25] used in this type of intervention [26], none have considered BCTs specifically within the context of digital interventions. As it cannot be assumed that BCTs have equal relevance to and effectiveness across different delivery methods, a review focused specifically on the role of BCTs in digitally delivered interventions for this population is a unique contribution to the literature.

### Objectives

The aim of this systematic review was three-fold: (1) to determine the effectiveness of exclusively digital diet and physical activity interventions to improve lifestyle behaviors or avoid excessive weight gain during pregnancy; (2) to investigate user engagement with the interventions; and (3) to identify and assess the usage of BCTs within the interventions.

## Methods

### Review Protocol

This systematic review and meta-analysis follows the Preferred Reporting Items for Systematic Reviews and Meta-Analysis (PRISMA) [27] and is registered with the International Prospective Register of Systematic Reviews (CRD42019124838; see Multimedia Appendix 1 for the PRISMA checklist).

### Search Strategy

A search of 6 databases (MEDLINE [Medical Literature Analysis and Retrieval System Online], PsycINFO, EMBASE, Cumulated Index to Nursing and Allied Health Literature [CINAHL] Plus, Web of Science, and ProQuest Dissertations and Theses) was conducted in February 2019 to identify relevant studies. Advanced searches of keywords and index terms covered 4 concept areas (pregnancy status, diet or physical activity intervention, digital technology, and study design) and were tailored according to each database (Multimedia Appendix 2). The Cochrane Library was also searched for related systematic reviews. Their reference lists along with those of eligible studies were hand searched. Once duplicates had been removed, 2 authors (AR and PC) independently screened and assessed each study for eligibility based on title and abstract.

### Inclusion and Exclusion Criteria

Studies were included in the study if they fulfilled the following population, interventions, comparators, and outcomes criteria.

#### Population

Pregnant women over the age of 18 years, of all BMIs, were included in this study. However, pregnant women with physical or mental health issues that would preclude them from participating in a diet- or physical activity–based intervention were excluded.

#### Interventions

Digital interventions targeting dietary behaviors or physical activity in pregnancy, with the aim of improving diet or physical activity during pregnancy or managing GWG, were included. Interventions aimed at increasing GWG were excluded. Interventions were exclusively digital and used text messages, apps or websites. Initial in-person or telephone study briefing sessions were deemed acceptable, as they seemed to reflect real-world situations in which health care professionals would introduce an intervention to pregnant women as a part of an antenatal care program. Interventions using interpersonal coaching or support beyond this were excluded, as were digital interventions delivered in a health care setting.

#### Comparators

Comparators were usual antenatal care, minimal interventions (ie, information only rather than active behavior change), or nondiet or physical activity interventions.

#### Outcomes

The primary outcomes were GWG (measured as total gain in kilos or compliance with IOM GWG guidelines [5]), changes in dietary behaviors, and changes in levels of physical activity. The secondary outcome was engagement, which was measured by intervention attrition rates and usage of the intervention features. BCTs were coded according to the BCT Taxonomy (version 1) [25].

### Study Design

Only randomized controlled trials (RCTs) and randomized pilot studies were included in this review.

#### Data Extraction and Data Synthesis

Data extracted for the systematic review included author and date of publication, geographical region, study design, behaviors targeted and specific behavioral goals, sample size, participant information, the technology used, intervention features, the theory used, gestational week in which intervention started, length of intervention, nature of control, attrition rate, engagement levels, outcome measures, and outcomes. Data extraction was completed independently by 2 authors (AR and HC). In addition, 2 authors (AR and PC) independently coded the BCTs within each intervention according to the BCT Taxonomy (version 1) [25]. If available and required, study development papers and protocols were retrieved for this purpose. In most instances, the authors were contacted for additional information. BCTs were coded only if there was unequivocal evidence of their existence [25]. Disagreements were discussed to reach consensus.

#### Quality Assessment

Two authors (AR and AS) independently evaluated the risk of bias within studies using the Cochrane Collaboration Risk of Bias (RoB) 2.0 tool for assessing the risk of bias (the Cochrane Collaboration) [28]. The 5 domains evaluated were risk of bias arising from the randomization process, risk of bias because of deviations from the intended interventions, missing outcome data, risk of bias in the measurement of the outcome, and risk of bias in the selection of the reported result. Bias was classified as low risk, high risk, or some concerns according to predetermined criteria set by RoB 2.0. Rating discrepancies among the authors were resolved through discussion. The risk of bias across studies could not be evaluated because of the small number of studies included in the meta-analyses [29].

### Data Analysis

Given the substantial heterogeneity of reported outcome measures in the identified studies, data could only be quantitatively pooled for meta-analysis from studies measuring GWG. Only 4 studies used intention-to-treat (ITT) analysis; therefore, separate analyses were conducted for ITT data and per-protocol (PP) data. Meta-analysis was used to determine the differences in mean total GWG (in kg) from baseline to postintervention using the inverse variance method. The odds ratio (OR) was meta-analyzed for studies reporting GWG as a dichotomous outcome (proportion of women exceeding IOM guidelines) using the Mantel-Haenszel method. The test for the overall pooled effect estimate was assessed using Z‐statistics at *P*=.04. Heterogeneity between studies was evaluated using the Cochran Q (chi-square test) and the *I^2^* statistics in the Review Manager 5.3 (the Cochrane Collaboration) [30]. Preplanned subgroup analyses were conducted comparing studies where BCTs could be identified in initial briefing sessions with those where none were apparent.

## Results

### Study Selection

**Figure 1:**
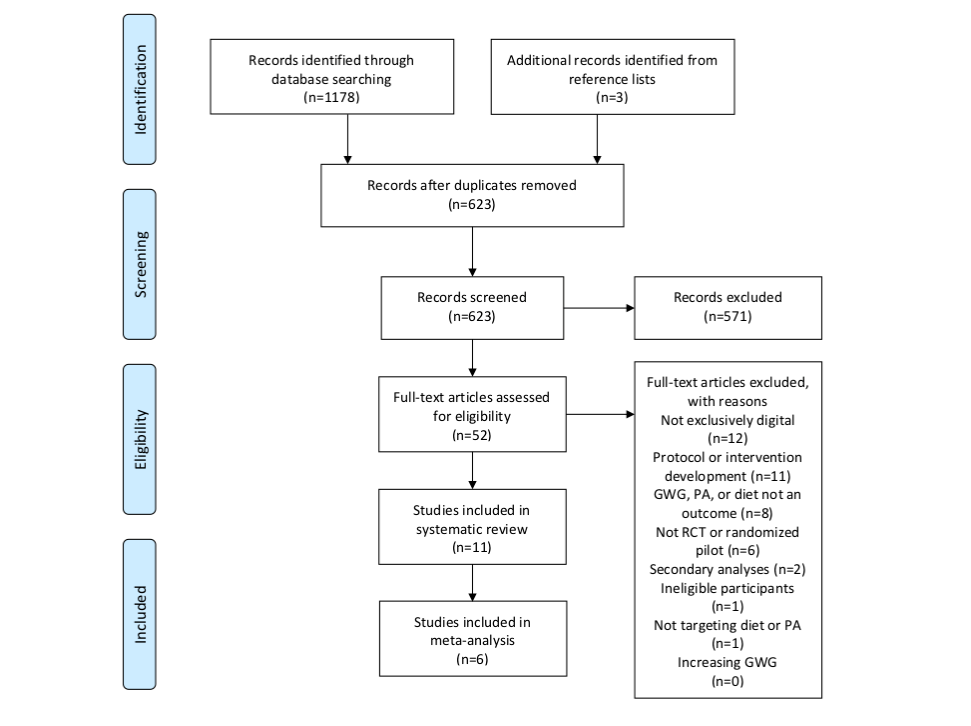
Study selection process.

The selection process is illustrated in Figure 1. A systematic search of 6 literature databases identified 623 nonduplicate study records. After the assessment of eligibility in accordance with the inclusion and exclusion criteria, 11 eligible studies were identified, of which 6 studies were included in subsequent meta-analyses.

### Study Characteristics

Table 1 summarizes the characteristics of the 11 studies included in this review. All studies were published between 2012 and 2019. Of the 11 studies, 7 were randomized pilot or feasibility studies [31-37] and 4 were RCTs [38-41]. Of the 4 RCTs, 2 reported being adequately powered [39,41], whereas the other 2 reported being underpowered as a result of a small starting sample [40] or low follow-up rate [38]. Overall, 9 studies took place in the United States [31-33,35,37-41] and 2 in Australia [34,36]. The sample sizes varied from 35 to 1689. In addition, 2 studies [31,38] targeted diet only, 4 studies [33,36,39,40] targeted physical activity only, and the remaining 5 studies [32,34,35,37,41] targeted both diet and physical activity. Seven studies reported on only one outcome: GWG [35,41], dietary [31,38], or physical activity [33,36,40] behaviors. The remaining 4 studies [32,34,37,39] reported on GWG, as well as changes in diet and physical activity. Three studies focused specifically on women with overweight or obesity [32,34,35], and 3 studies [33,39,40] focused on inactive or sedentary women.

The delivery method varied across studies with 4 using text messaging [31,32,38,40], 3 using an app [33,35,37], 3 using a website [36,39,41], and 1 combining text messaging with a website [34]. In total, 7 studies included an interpersonal briefing session at the start of the study [31-34,37,39,40]. In 4 studies [31,32,37,40], these sessions were for screening or study measures only, but 3 studies [33,34,39] included discussions with intervention participants about the intervention features. In one study [39], these discussions involved an in-person tutorial on how to use the website and its features and practice tracking physical activity. In another study [34], the researcher discussed individual GWG targets and weight monitoring and asked participants to set a physical activity or dietary goal. In a third study [33], a 30-min in-person session covered physical activity recommendations, goal setting, problem solving, social support, and planning for lapses.

**Table 1:** Study characteristics.

Authors, year	Country	Study design	Sample size, n	Technology used	Behaviors targeted	Outcomes measured	Participants
Evans et al, 2012 [31]	United States	Pilot	90	Text	Diet	Diet	Low-income, underserved pregnant women
Pollak et al, 2014 [32]	United States	Pilot	35	Text	Diet and physical activity	GWG^a^, diet and physical activity	BMI 25-40 kg/m^2^; gestation 12-21 weeks
Evans et al, 2015 [38]	United States	RCT^b^	996	Text	Diet	Diet	Military women; gestation <14 weeks
Smith et al, 2016 [39]	United States	RCT	51	Website	Physical activity	GWG, diet, and physical activity	Sedentary women; gestation 10-14 weeks
Choi et al, 2016 [33]	United States	Pilot	30	App	Physical activity	Physical activity	Physically inactive women; gestation 10-20 weeks
Willcox et al, 2017 [34]	Australia	Pilot	91	Text and website	Diet and physical activity	GWG, diet, and physical activity	BMI>25 kg/m^2^; gestation 10-17.6 weeks
Redman et al, 2017 [35]	United States	Pilot	54	App	Diet and physical activity	GWG	BMI>25 kg/m^2^; gestation 10.4-13.6 weeks
Hayman et al, 2017 [36]	Australia	Pilot	77	Website	Physical activity	Physical activity	Gestation 10-20 weeks
Huberty et al, 2017 [40]	United States	RCT	80	Text	Physical activity	Physical activity	Not meeting physical activity recommendations; gestation 8-16 weeks
Olson et al, 2018 [41]	United States	RCT	1689	Website	Diet and physical activity	GWG	BMI 18.5-35 kg/m^2^; gestation <20 weeks
Dahl et al, 2018 [37]	United States	Pilot	87	App	Diet and physical activity	GWG	BMI≥18.5 kg/m^2^; gestation <20 weeks

^a^GWG: gestational weight gain.

^b^RCT: randomized controlled trial.

### Risk of Bias

Table 2 summarizes the study quality assessment. The overall study quality was variable. Five studies were deemed to have an overall *high risk* of bias, 3 had a *low risk* of bias, and 3 were classified as having *some concerns*.

**Table 2:** Risk of bias summary.

Study, year	Domain 1: risk of bias arising from the randomization process	Domain 2: risk of bias because of deviations from the intended interventions	Domain 3: missing outcome data	Domain 4: risk of bias in the measurement of the outcome	Domain 5: risk of bias in selection of the reported result	Overall risk of bias
Evans et al, 2012 [31]	High	High	High	Some concerns	Low	High
Pollak et al, 2014 [32]	High	Low	Low	Some concerns	Low	High
Evans et al, 2015 [38]	Low	Low	Low	Some concerns	Low	Some concerns
Smith et al, 2016 [39]	Low	High	High	High	Low	High
Choi et al, 2016 [33]	Low	Low	Low	Low	Low	Low
Willcox et al, 2017 [34]	Low	Some concerns	Low	Some concerns	Low	Some concerns
Redman et al, 2017 [35]	Low	Low	Low	Low	Low	Low
Hayman et al, 2017 [36]	Low	High	High	Low	Low	High
Huberty et al, 2017 [40]	Some concerns	Low	Low	Low	Low	Some concerns
Olson et al, 2018 [41]	Low	Low	Low	Low	Low	Low
Dahl et al, 2018 [37]	Some concerns	High	High	Some concerns	Low	High

### Description of the Interventions

Multimedia Appendix 3 summarizes the intervention features, outcome measures, effectiveness, attrition, and engagement data. All interventions were theory-based, with social cognitive theory [42] being the most widely used (n=8). All trials started in the first or second trimester of pregnancy. The study duration varied considerably with one trial lasting 4 weeks [36], 2 trials lasting 12 weeks [33,37], one trial lasting 16 weeks [32], and the remaining trials lasting 20 weeks or more, completing at or close to term. Most studies compared interventions with usual care [31,32,34,35,38,39] or access to information-only aspects of the intervention [33,36,40,41]. In one study, the control was an equivalently structured intervention targeting stress reduction [37].

### Effectiveness of Interventions

Of the 11 studies, 3 reported significant positive effects of their interventions on GWG [34,35] and physical activity [34,36] in comparison with control groups.

**Figure 2:**
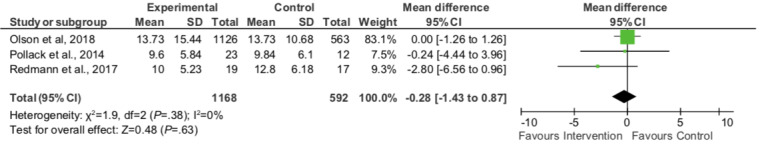
Pooled analysis of digital interventions on total gestational weight gain (kg)—intention-to-treat studies.

**Figure 3:**
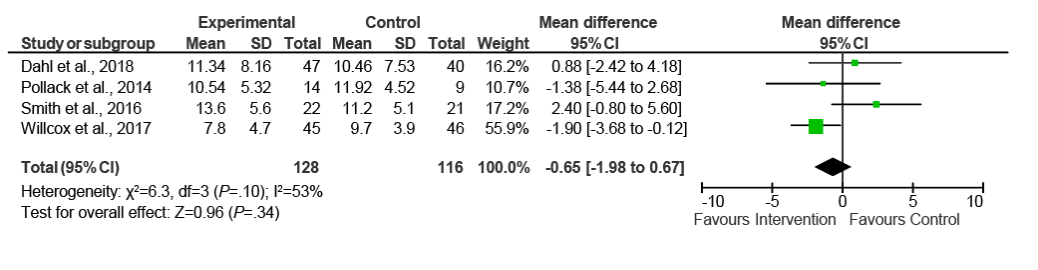
Pooled analysis of digital interventions on total gestational weight gain (kg)—per-protocol studies.

**Figure 4:**
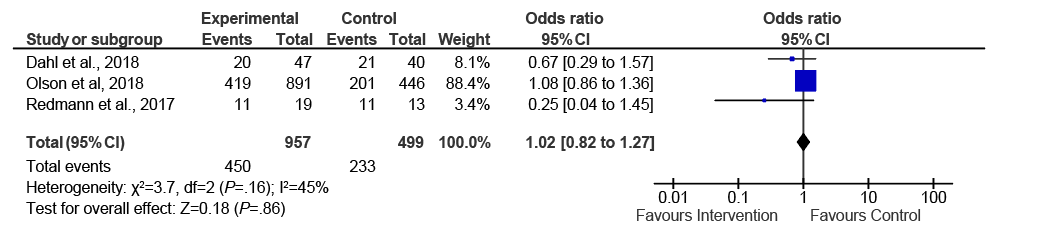
Pooled analysis of digital interventions on the percentage of women exceeding Institute of Medicine guidelines—per-protocol studies.

The 6 studies with GWG as the primary outcome varied in their measurement of total GWG. Three studies used the difference between last measured weight before delivery (34-37 weeks) and baseline weight (10-17 weeks) [34,35,41]. Two studies [37,39] used self-reported prepregnancy weight as the starting weight, and one study [32] used a model of estimated mean weights at 16 and 40 weeks. One study showed significantly lower total GWG among intervention participants [34], whereas another showed significantly fewer intervention participants exceeding the IOM guidelines [35]. The remaining studies [32,37,39,41] showed no significant difference between the intervention and control groups on any GWG measures. A meta-analysis of ITT data (n=3) showed a nonsignificant effect of the interventions, with a mean difference in total GWG of −0.28 kg (95% CI −1.43 to 0.87) using the inverse variance method and a fixed effects model (*I^2^*=0%; *P*=.38; Figure 2). The mean difference in total GWG for PP data (n=4) was −0.65 kg (95% CI −1.98 to 0.67; *I^2^*=53%; *P*=.10; Figure 3). The subgroup analyses revealed no significant change in this result (Multimedia Appendix 4). A meta-analysis of studies reporting PP percentages exceeding IOM guidelines showed no effect of interventions relative to comparators (OR 1.02, 95% CI 0.82-1.27; *I^2^*=45%; *P*=.16; Figure 4).

Of the 7 studies reporting physical activity, 3 showed significant positive effects of the intervention on levels of physical activity [34,36,39]. Of these, one study relied on self-reported physical activity and showed significantly smaller reductions in total, light-intensity, and moderate-intensity physical activities in the intervention group compared with the control group [34]. Two other studies used smart technology to provide an objective measure of physical activity (Fitbit [36] and SenseWear Mini Arm Band [39]). One study reported a significant increase in moderate-to-vigorous physical activity in the intervention group compared with the control group, although over a 4-week period only [36]. Another study reported significantly greater levels of sustained physical activity for intervention participants compared with control participants in midpregnancy, but the effect had disappeared by the end of the intervention [39]. Only one of the 6 studies reporting on dietary behaviors was effective in improving diet [37]. Using a self-report measure (the Rapid Eating and Activity Assessment for Participants Short Scale), intervention participants in this study scored significantly higher on healthy eating practices (measuring meal behaviors and serving frequencies) compared with the control participants.

### BCTs

Multimedia Appendix 5 summarizes the 23 different BCTs identified within the interventions. Only one study specified all the included BCTs [34], and this study reported the highest number of BCTs (n=17). In 2 interventions, only 1 BCT was evident [31,38]. In the remaining interventions, the number of BCTs ranged from 5 to 15. The 3 effective interventions used, on average, twice the number of BCTs compared with other interventions (mean 14, SD 2.9 vs mean 6.8, SD 4.1). Information about health consequences was the only BCT to be used in all interventions. Beyond this, goal setting (behavior) appeared in 8 interventions and problem solving and self-monitoring (behavior and outcome) in 7 interventions. Seven BCTs were common to the 3 interventions, showing a significant effect [34-36]. These were goal setting (behavior), problem solving, review of behavior goals, feedback on behavior, social support, information about health consequences, and information about emotional consequences. Review of the behavior goal was the only BCT used exclusively in the 3 effective interventions. The 3 information-only interventions that included no active or interactive BCTs, such as goal setting, self-monitoring, problem solving, or feedback, were ineffective [31,38,40].

### Engagement With Interventions

Attrition rates were reported by all the studies, although 2 studies [31,40] only provided figures for all participants rather than separating intervention and control participants (Multimedia Appendix 3). Six studies reported intervention attrition rates of 10% or less [33-35,39-41]. These studies identified the reasons for dropping out of the study by distinguishing between medical and study-related reasons. Three of these studies [34,35,40] incentivized participants and 2 [33,39] introduced an element of self-selection by recruiting women who were motivated or willing to increase their physical activity. In the remaining 5 studies, intervention attrition was more than 30% and lost to follow-up reasons were not explained beyond being unable to recontact participants.

Six studies [32-36,41] reported intervention engagement levels using a variety of measures, including usage of self-monitoring, goal setting, action planning, and social media features, response to texts, completion of tasks, and website logins (Multimedia Appendix 3). Four studies [32,34,36,41] evaluated participants’ views of the intervention, although only one study [36] explored the user experience of the technology. Four studies [32,34-36] reported engagement levels over 70%, including the 3 interventions with significant effects [34-36]. A further study [33] started with a similarly high level of engagement, although it fell to below 50% over the course of the 12-week intervention. The final study [41] reported an engagement level of 46%. Five studies [32-36] integrated interactive elements to encourage engagement with the intervention. Two interventions [32,34] sent 4 or more text messages per week, encouraging self-monitoring and giving tailored feedback, and 3 studies [33,35,36] used in-app messaging to provide tailored feedback.

## Discussion

### Principal Findings

The aim of this systematic review was to determine the effectiveness of diet and physical activity interventions during pregnancy delivered using exclusively digital technology, and to provide insight into how BCTs and engagement with intervention features might be driving effectiveness. A total of 11 studies were identified, all of which were published from 2012 onward, with 6 studies published in 2017 and 2018. App and mobile-accessible website interventions appeared only in the last 2 years, reflecting the emergent nature of mobile health interventions to encourage healthy behaviors during pregnancy. Meta-analyses showed no significant benefit of exclusively digital interventions on total GWG. Substantial heterogeneity in outcome measures of change in dietary behaviors and physical activity precluded further meta-analyses. BCT coding identified 7 BCTs that were common to all effective interventions. Effective interventions averaged over twice as many BCTs from the *goals and planning* and *feedback and monitoring* domains as ineffective interventions. Six studies reported on user engagement, and their data indicated a positive association between high engagement with key BCTs and greater intervention effectiveness. Interventions using proactive messaging, such as reminding participants to engage in BCTs or providing feedback or tips, appeared to have higher levels of engagement.

### Effectiveness

Meta-analyses of the digital interventions measuring GWG showed no effect on the total GWG or weight gain within the IOM guidelines. Although the majority of these studies were pilot RCTs and insufficiently powered to detect an effect, these findings indicate that exclusively digital interventions to manage GWG may be less effective than those using interpersonal delivery. Lack of consistency in outcome measures precluded meta-analyses of the effects of digital interventions on dietary behaviors and physical activity. Only 3 of the 7 studies measuring changes in physical activity reported significant effects of the intervention, suggesting that for physical activity interventions during pregnancy, digital delivery may similarly be less effective than interpersonal delivery [43].

The 11 interventions varied considerably in terms of not only the targeted behaviors but also the technologies, functionalities, and BCTs used. As such, it would be premature to conclude that exclusively digital delivery methods *per se* are less effective than interpersonal delivery methods for lifestyle interventions during pregnancy. Indeed, one of the included studies made a direct comparison of digital delivery and in-person delivery of the same intervention [35]. It found the intervention to be effective via both delivery methods, with digital delivery showing greater adherence and lower costs (for both participants and clinics) compared with in-person delivery.

### BCTs

This systematic review aimed to identify the BCTs associated with effective interventions. The number of identifiable BCTs ranged from 1 to 17, with the 2 most effective interventions [34,35] using the highest number (n=17 and n=15). The average number of BCTs was 9 compared with approximately 5 reported in 2 earlier systematic reviews of lifestyle interventions targeting pregnant women [44,45]. It is unclear whether this increase reflects a trend toward greater intervention complexity, reflects the opportunity digital interventions afford to include more components, or is simply a matter of improved reporting of BCTs. Consistent with previous systematic reviews, this review found that effective interventions tended to report a greater number of BCTs [44,46]. A meta-analysis of 122 physical activity and healthy eating interventions (for all adults) showed effectiveness to be a function of not simply the number of BCTs but particular BCTs—*self-monitoring* and at least one other technique derived from control theory [47]. In this review, *self-monitoring* appeared in 7 interventions but was notably absent from one of the 3 interventions showing a significant effect [36]. The 3 effective interventions did however average over twice as many *goals and planning* and *feedback and monitoring* BCTs as ineffective interventions (mean 7.6, SD 2.1 and mean 3.4, SD 2.9, respectively).

There was considerable variation in the execution and delivery of BCTs. For example, in some studies, participants were invited to set a single goal, whereas in others, they were able to set multiple goals. In some instances, participants were encouraged to choose their own goal, whereas in others, the goal was prescribed. Similarly, some interventions required participants to submit self-monitoring data regularly, whereas others recommended and provided functionality for self-monitoring but did not make it obligatory. Four studies proactively messaged participants to remind them to self-monitor [32-34,37], whereas one messaged participants only if they failed to self-monitor [35]. In 3 of the studies that incorporated an initial in-person session for intervention participants [33,34,39], one or more BCTs were identifiable at this stage, raising the question as to whether the content of these sessions contained sufficient BCTs in their own right to bring about a change. The influence of these variations in the context, execution, and delivery of BCTs on intervention effectiveness could not be quantified by the methods used in this study. Given the interactive and dynamic nature of digital interventions, additional measures may be needed to capture the impact of features, such as the timing of delivery and degree of individual tailoring of BCTs.

Consistent with other systematic reviews of this type of intervention [44,48,49], *information about health consequences* was the most widely used BCT, which featured in all interventions. *Goal setting (behavior)* was the next most widely used BCT, appearing in all but 3 text message–only interventions [31,38,40]. *Problem solving,* *self-monitoring of behavior, self-monitoring of outcomes,* and *instructions on how to perform a behavior* all appeared in 7 interventions. *Feedback on behavior* was provided in 6 interventions, including the 3 [34-36] reporting significant effects of the interventions. The BCT *review behavior goal* was only present in the 3 effective interventions [34-36], suggesting that this may be a critical active ingredient in these digital interventions. It is possible that *review behavior goal* in combination with *self-monitoring of behavior* and *feedback on behavior* work together to support the self-regulation of energy balance behaviors during pregnancy.

*Social support* was present in 6 interventions [33-37,40], including the 3 effective interventions. Once again, the execution of *social support* varied, ranging from advice on how to seek support to online group forums for participants. There is no consensus on whether social support or interaction with other participants improves intervention effectiveness, and no clear pattern emerged from this review [14,23,50]. More research is needed to understand the type of social support that is most beneficial to digital interventions encouraging healthy behaviors during pregnancy.

Insufficient description of intervention components, coupled with a lack of systematic recording of BCTs, compromised the quality of the BCT analysis. Only 1 study provided details of all the BCTs [34] used in the intervention, whereas the presence of BCTs had to be inferred from descriptions of the interventions in all other studies. This raises the possibility that there may be additional but unreported BCTs in other studies. Previous studies that have coded BCTs used in gestational weight management trials have called for greater clarity and accuracy in the reporting of BCTs [26]. Without systematic reporting of active intervention ingredients, it is difficult to precisely determine which BCTs may be driving effectiveness.

### Engagement

Six studies provided measures of user engagement [32-36,41]. These varied considerably, including the number of replies to texts, frequency of inputting weight monitoring data and logging onto and viewing web pages. Only one study [36] provided feedback on user experience. Given the importance of user engagement to the success or otherwise of self-managed digital interventions, more detailed and standardized measures could facilitate better evaluation and cross-study comparison [51]. Perski et al [52] proposed more comprehensive measures, including both the extent (ie, amount, frequency, duration, depth) of usage and the user experience. Reinforcing the need for a more holistic evaluation of engagement, Yardley et al [24] proposed identifying and reporting on *effective engagement* rather than simply higher levels of engagement. The combination of web analytics and survey feedback clearly offers the opportunity to develop specific and relevant indices of engagement [53].

The 3 effective interventions [34-36] all reported engagement levels of over 70% with key BCTs (*goal setting* [34,36], *self-monitoring* [34,35], and *action planning* [36]). Conversely, the study with the lowest engagement level, where only 46% of participants logged onto the website at least once every 45 days, and the use of *goal setting* and *self-monitoring* features was 35% and 23% of the participants respectively, reported no effect of the intervention [41]. These findings suggest that ineffectiveness may be partially a function of poor engagement with key BCTs rather than poorly designed interventions *per se*. Supporting this hypothesis, this study with low levels of engagement [41] conducted secondary analyses investigating whether usage patterns of the intervention features reduced the risk of excessive GWG and found frequent usage patterns were associated with lower total GWG [54,55]. In addition, the use of the dietary tool (*goal setting* and *self-monitoring*) was associated with improved GWG management for women with normal BMI, although not for those with high BMI.

One consistent feature of the interventions reporting the levels of engagement over 70% was regular in-app messaging or text messaging giving encouragement, reminders to self-monitor, or tailored feedback on progress. Prompts and reminders have been shown to promote engagement in digital interventions [56]. Similarly, tailoring messages to the characteristics and usage patterns of the individual has been shown to improve adherence [57]. Notably, the study [41] in which participants were sent a generic weekly email reported particularly low levels of engagement. The frequency and timing of these messages are also important [58]. One study [34] delivering 4 to 5 texts per week found that 79% of participants thought the frequency of messages was *about right*, although 21% thought it was *too high*. Another study [40] investigated the dose and timing of messages to promote physical activity by comparing 3 texts per week with daily texts and found daily texts to be less effective, indicating that too much messaging can be counterproductive. None of the studies referred to the use of gamification techniques to promote engagement, although elements of some of the interventions could potentially be classified as gamification, such as team challenges [37,59]. Incorporating gamification features, such as badges and challenges, has been shown to increase regular engagement and immersion in digital health interventions [60,61].

The final issue regarding engagement concerns who the interventions are reaching. Only one study reported (in follow-up analyses) on high versus low engagers, revealing significant differences according to ethnicity, income group, BMI, and partner status [54]. Often, it is those who would benefit most from behavior change who are least likely to engage in behavior change interventions [62]. Greater insight into who engages with the interventions could enhance learnings from these studies.

### Strengths and Limitations

A strength of this systematic review is that it is the first to focus on exclusively digital interventions to promote healthy dietary behaviors, physical activity, or weight management during pregnancy. In addition to evaluating their effectiveness, this review conducted a rigorous assessment of BCTs and participant engagement to provide detailed insight into what may be driving effectiveness—a crucial step if the cost and reach advantages of digital interventions are to be leveraged. However, there are several limitations to this systematic review. First, most of the studies included were pilot studies rather than RCTs and, as such, were not adequately powered to show effect sizes. Moreover, there was considerable heterogeneity of intervention features and outcome measures, and several studies reported the results from PP analyses rather than ITT. As such, the results of the meta-analyses should be interpreted with caution. Second, the risk of bias across the studies was moderate to high, with 5 studies scoring overall *high* and a further 3 scoring as *some concerns*, as assessed by RoB 2.0. Third, the timing of the interventions within pregnancy varied both in terms of the start point within the gestational window and duration. This, coupled with inconsistent measures of GWG and, in some cases, reliance on self-reported weight measures should be considered when appraising the findings. Finally, limited reporting of intervention features meant that not all BCTs were recorded. Providing more detailed descriptions of the interventions’ design and content (in supplementary files) would augment shared learnings from these studies. Similarly, more detailed and consistent engagement measures would have enhanced the interpretation of user engagement data.

### Conclusions

Meta-analyses show that the mean impact on GWG of exclusively digital interventions targeting dietary behaviors, physical activity, and weight management during pregnancy to be nonsignificant, meaning that the current exclusively digital interventions are less effective than interpersonal interventions in this field. There was considerable variation in intervention effectiveness across the 11 studies, with 3 studies from 2017 reporting significantly positive effects of their interventions. Limited data precluded confident identification of the ingredients of successful interventions, although this review suggests that variation in effectiveness could be partially explained by the BCTs used and levels of interactivity to encourage engagement with the intervention features. Effective interventions used more BCTs (particularly BCTs from *goals and planning* and *feedback and monitoring* domains) and reported higher levels of engagement with key BCTs. Effective interventions also used interactivity, in the form of messages of encouragement, personalized feedback, and prompts to remind participants to use key BCTs, such as *goal setting* and *self-monitoring,* to promote engagement.

There are several compelling reasons for considering using digital interventions to promote healthy energy balance behaviors during pregnancy: smartphone ownership is over 90% among women of childbearing age [63] and usage of pregnancy apps is pervasive [64]; digital interventions have broader reach and lower costs than interpersonal interventions [35,65]; and apps have been shown to be particularly successful in reaching those who may be less likely to engage with traditional antenatal health care [66]. Meanwhile, midwives frequently report that they have neither the time nor expertise to advise pregnant women on physical activity or healthy eating [67]. Future research needs to consider how to seize the opportunities presented by new technologies to enhance interactivity, improve user engagement, and bring greater effectiveness to these digital interventions.

## Appendix

Multimedia Appendix 1 PRISMA Checklist.

Multimedia Appendix 2 Subject headings and key words for search.

Multimedia Appendix 3 Intervention features, engagement and effectiveness.

Multimedia Appendix 4 Subgroup analyses.

Multimedia Appendix 5 Summary of BCTs.

## Abbreviations

BCT: behavior change technique
GWG: gestational weight gain
IOM: Institute of Medicine
ITT: intention-to-treat
OR: odds ratio
PP: per-protocol
PRISMA: Preferred Reporting Items for Systematic Reviews and Meta-Analysis
RCT: randomized controlled trial
