# Supplementary material for: Exclusively Digital Health Interventions Targeting Diet, Physical Activity, and Weight Gain in Pregnant Women: Systematic Review and Meta-Analysis
Source: JMIR Mhealth Uhealth. 2020 Jul 10;8(7):e18255. doi: 10.2196/18255 (PMC7382015; doi:10.2196/18255)
Supplement: Multimedia Appendix 2 [file mhealth_v8i7e18255_app2.pdf]

Multimedia Appendix 2: Subject headings and key words for search.

1. Example of full electronic search strategy (PsychInfo)

1. (pregnan* or gestation* or matern* or antenatal or ante-natal or prenatal or pre-natal or perinatal or peri-natal).mp. [mp=title, abstract, original title, name of substance word, subject heading word, floating sub-heading word, keyword heading word, organism supplementary concept word, protocol supplementary concept word, rare disease supplementary concept word, unique identifier, synonyms]

2. pregnancy/

3. prenatal care/

4. 1 or 2 or 3

5. (diet* or healthy eating or nutrition or lifestyle or life style or physical activity or physical exertion or exercise or activ* fitness or weight gain or weight manag* or weight control).mp. [mp=title, abstract, original title, name of substance word, subject heading word, floating sub-heading word, keyword heading word, organism supplementary concept word, protocol supplementary concept word, rare disease supplementary concept word, unique identifier, synonyms]

6. physical activity/

7. exercise/

8. lifestyle/ or healthy lifestyle/

9. diet therapy/

10. body weight control/

11. body weight gain/

12. 5 or 6 or 7 or 8 or 9 or 10 or 11

13. 4 and 12

14. (((((app or apps or mobile app* or ehealth or e-health or mhealth or mobile health or m-health or internet or web or technology or smartphone or smart phone or smart-phone or cellphone or cell-phone or cell phone or mobile) adj phone) or online or on-line or text) adj messag*) or SMS or telehealth).mp. [mp=title, abstract, original title, name of substance word, subject heading word, floating sub-heading word, keyword heading word, organism supplementary concept word, protocol supplementary concept word, rare disease supplementary concept word, unique identifier, synonyms]

15. text messaging/

16. mobile application/

17. internet/

18. mobile phone/

19. e-mail/

20. 14 or 15 or 16 or 17 or 18 or 19

21. 13 and 20

22. limit 21 to (human and english language)

23. controlled clinical trial/

24. feasibility study/

25. pilot study/

26. health care survey/ or health survey/

27. (random* control* or feasibility or pilot or survey).mp. [mp=title, abstract, original title, name of substance word, subject heading word, floating sub-heading word, keyword heading word, organism supplementary concept word, protocol supplementary concept word, rare disease supplementary concept word, unique identifier, synonyms]

28. 23 or 24 or 25 or 26 or 27

29. 22 and 28

2. Search terms for each database

Medline

<u>Concept 1:</u>
Keywords: pregnan* or gestation* or matern* or antenatal or ante-natal or prenatal or pre-natal or perinatal or peri-natal
MeSH: Pregnancy or Pregnant Women; Prenatal Care

<u>Concept 2:</u>
Keywords: diet* or healthy eating or nutrition or lifestyle or life style or physical activity or physical exertion or exercise or activ* fitness or weight gain or weight manag* or weight control
MeSH: Exercise; Life Style; Healthy Lifestyle; Body Weight; Weight Gain; Nutrition Therapy; Diet Therapy or Diet or Healthy Diet

<u>Concept 3:</u>

Keywords: app or apps or mobile app* or ehealth or e-health or mhealth or mobile health or m-health or internet or web or technology or smartphone or smart phone or smart-phone or cellphone or cell-phone or cell phone or mobile adj phone or online or on-line or text adj messag* or SMS or telehealth
MeSH: Telemedicine; Mobile Applications; Cell Phone; Text Messaging; Internet
Embase

Concept 1:
Keywords: pregnan* or gestation* or matern* or antenatal or ante-natal or prenatal or pre-natal or perinatal or peri-natal
MeSH: Pregnancy or Pregnant Women; Prenatal Care

Concept 2:
Keywords: diet* or healthy eating or nutrition or lifestyle or life style or physical activity or physical exertion or exercise or activ* fitness or weight gain or weight manag* or weight control
MeSH: Physical activity, Exercise, Lifestyle or Healthy Lifestyle, Diet or Diet Therapy, Body Weight Gain, Body Weight Control

Concept 3:
Keywords: app or apps or mobile app* or ehealth or e-health or mhealth or mobile health or m-health or internet or web or technology or smartphone or smart phone or smart-phone or cellphone or cell-phone or cell phone or mobile adj phone or online or on-line or text adj messag* or SMS or telehealth
MeSH: Mobile Application; Mobile Phone; Text Messaging; Internet; e-mail

PsycINFO

Concept 1:
Keywords: pregnan* or gestation* or matern* or antenatal or ante-natal or prenatal or pre-natal or perinatal or peri-natal
MeSH: Pregnancy; Prenatal Care

Concept 2:
Keywords: diet* or healthy eating or nutrition or lifestyle or life style or physical activity or physical exertion or exercise or activ* fitness or weight gain or weight manag* or weight control
MeSH: Physical Activity; Exercise; Lifestyle or Lifestyle Changes; Diets; Body Weight; Weight Gain; Weight Control; Health Behaviour; Behaviour Change

Concept 3:
Keywords: app or apps or mobile app* or ehealth or e-health or mhealth or mobile health or m-health or internet or web or technology or smartphone or smart phone or smart-phone or cellphone or cell-phone or cell phone or mobile adj phone or online or on-line or text adj messag* or SMS or telehealth.
MeSH: Mobile Devices or Computer Applications; Cellular Phones; Text Messaging; Internet; Electronic Communication or Computer Mediated Communication

CINAHL PLUS

Concept 1:
Title or Abstract: pregnan* or gestation* or matern* or antenatal or ante-natal or prenatal or pre-natal or perinatal or peri-natal

Concept 2:

All text: diet* or “healthy eating” or nutrition or lifestyle or “life style” or “physical activity” or “physical exertion” or exercise or “activ* fitness” or “weight gain” or “weight manag*” or “weight control”

Concept 3:
All text: app or apps or mobile app* or ehealth or e-health or mhealth or “mobile health” or m-health or internet or web or technology or smartphone or “smart phone” or smart-phone or cellphone or cell-phone or “cell phone” or “mobile phone” or online or on-line or “text messag*” or SMS or telehealth

Concept 4:
“random* control*” or “control* trial” or pilot or feasibility or survey

Web of Science

Concept 1:
TS= (pregnan* or gestation* or matern* or antenatal or ante-natal or prenatal or pre-natal or perinatal or peri-natal)

Concept 2:
TS= (diet* or “healthy eating” or nutrition or lifestyle or “life style” or “physical activity” or “physical exertion” or exercise or “activ* fitness” or “weight gain” or “weight manag*” or “weight control”)

Concept 3:
TS= (app or apps or mobile app* or ehealth or e-health or mhealth or “mobile health” or m-health or internet or web or technology or smartphone or “smart phone” or smart-phone or cellphone or cell-phone or “cell phone” or “mobile phone” or online or on-line or “text messag*” or SMS or telehealth)

Concept 4:
TS=(“random* control*” or “control* trial” or pilot or feasibility or survey)

ProQuest Dissertations & Theses Global

Concept 1:
Abstract: pregnan* or gestation* or matern* or antenatal or ante-natal or prenatal or pre-natal or perinatal or peri-natal

Concept 2:
Abstract: diet* or “healthy eating” or nutrition or lifestyle or “life style” or “physical activity” or “physical exertion” or exercise or “activ* fitness” or “weight gain” or “weight manag*” or “weight control”

Concept 3:
Abstract: app or apps or mobile app* or ehealth or e-health or mhealth or “mobile health” or m-health or internet or web or technology or smartphone or “smart phone” or smart-phone or cellphone or cell-phone or “cell phone” or “mobile phone” or online or on-line or “text messag*” or SMS or telehealth

Concept 4:
Anywhere:“random* control*” or “control* trial” or pilot or feasibility or survey
