# Supplementary material for: Exclusively Digital Health Interventions Targeting Diet, Physical Activity, and Weight Gain in Pregnant Women: Systematic Review and Meta-Analysis
Source: JMIR Mhealth Uhealth. 2020 Jul 10;8(7):e18255. doi: 10.2196/18255 (PMC7382015; doi:10.2196/18255)
Supplement: Multimedia Appendix 3 [file mhealth_v8i7e18255_app3.pdf]

Multimedia Appendix 3: Intervention features, engagement and effectiveness.

Study	Theory used	Intervention objective	Specific behaviour(s) targeted	Outcome measure(s)	Start (and length) of intervention	Control	Intervention features	Incentivisation	Intervention attrition	Engagement	Analysis	Results
Evans et al 2012	Social Cognitive Theory, the Transtheoretical Model and the Health Belief Model	To change health, health care beliefs, practices and behaviours of pregnant women	Fruit and vegetable consumption	1)24-item telephone survey, self-reported behaviour	NA	Standard care	Text messages – approx. 3 per week	None	30.1%*	NA	PP	Ineffective – no differences in fruit and vegetable consumption
Pollak et al 2014	Social Cognitive Theory	To promote healthy GWG amongst overweight and obese women	Diet and exercise	1)GWG – clinical weight between baseline and delivery 2)Pregnancy Physical Activity Questionnaire 3)PRIME	Gestation 12-21 weeks (~16 weeks)	Standard text messages	Tailored text messages; goal setting; weight and behaviour monitoring; feedback	Women who responded to at least 80% of texts entered into a raffle for $25 gift card	39.1%	86% read and responded to texts about self-monitoring behavioural goals and GWG	ITT & PP	Ineffective - ITT: no differences in GWG PP: non-significant difference in GWG. No differences in

				screen (18 questions on food consumption)								diet or physical activity
Evans et al 2015	Social Cognitive Theory, Transtheoretical Model and Health Belief Model	To increase health-promoting behaviours and decrease risk behaviours and determine whether there is a dose-response effect	Fruit and vegetable consumption	1)24 item on-line survey, self-reported behaviour	Gestation <14 weeks (~26 weeks, to term)	Usual care only	Text messages – approx. 3 per week	None	51.4%	NA	PP	Ineffective– no differences in fruit and vegetable consumption
Smith et al 2016	Social Cognitive Theory	To prevent excessive GWG by increasing	Physical activity	1)GWG – difference between last weight measure by research staff at 34-36	Gestation 10-14 weeks (~23 weeks, to 34-36 weeks)	Website with general prenatal diet and PA recs	Control website plus goal setting, problem solving, journal, calendar,	None	7.7%	NA	PP	Ineffective – no differences in GWG, but significantly greater

		physical activity		weeks and self-reported pre-pregnancy weight and compliance with IOM guidelines 2)Sense Wear Mini armband and self-reported record of physical activity when armband not worn) 3)3-day diet record		only (NB no info on GWG)	community forum					physical activity and energy intake mid-pregnancy for intervention group, but no differences by end
Choi et al 2016	Social Cognitive Theory	To increase physical activity	Steps	1)Daily step count measure by Fitbit	Gestation 10-20 weeks (12 weeks)	Fitbit only (with instruction to increase steps)	Fitbit, app with daily messages, tips, problem solving techniques, modelling, activity diary, feedback and self-monitoring	None	6.7%	Week 1:76% and 81% response to daily messages and activity diary Week 12: 50% and 40%	ITT	Ineffective – non-significant increase in daily steps and lower perceived barrier to being active for intervention group

Willcox et al 2017	Social Cognitive Theory	To promote healthy diet, physical activity and GWG within guidelines	Diet and physical activity	1)GWG - difference between baseline and 36 week visit and compliance with IOM guidelines 2)Food frequency questionnaire 3)Pregnancy Physical Activity Questionnaire	Gestation 10-17.6 weeks (~20 weeks, to 36 weeks)	Usual care	Tailored text messages, video messages, chat room interaction, goal setting, weight monitoring (multiple modalities)	A$20 shopping voucher on completion of evaluation at base line and 36 weeks (all participants) and A$20 iTunes voucher to compensate for data usage (intervention participants)	10%	98% read all or most texts; 96% replied; 95% set goals; 83% self-monitored GWG; 31% joined social media; 0.87 average per month website visits	PP	Effective - significantly lower GWG and less likely to reduce total physical activity in intervention group. No difference in % exceeding GWG guidelines or dietary behaviour
Redman et al 2017	Theory based, but non-specific	To decrease the proportion of women who excess IOM guidelines	Calorie intake Steps	1)GWG – difference between weight at initial (10-13 weeks) and final (35-36) study visits and compliance with IOM guidelines	Gestation 10.4-13.6 weeks (~24 weeks, to 35-36 weeks)	Usual care	Weekly/biweekly app-based coaching on diet, physical activity and weight, daily self-monitoring of diet, steps and weight	None	0%	71% submitting weight and 73% steps data	ITT & PP	Effective - significantly lower % exceeding GWG guidelines and average GWG non-significantly

												lower in intervention group
Hayman et al 2017	Social Cognitive Theory	To promote physical activity	Physical activity	1)GeneActiv accelerometer, measuring moderate to vigorous physical activity	Gestation 10-20 weeks (4 weeks)	Information/ guidelines only	Four weekly modules: action planning tool; feedback messages	None	30.8%	99% completion of weekly modules; 72% goal setting and action planning tasks	PP	Effective – significant positive difference in moderate to vigorous physical activity in intervention group
Huberty et al 2017	Social Cognitive Theory, the Transtheoretical Model and the Health Belief Model	To improve physical activity	Activity and sedentary behaviour	1)Fitbit, measuring sedentary, light, fairly active and very active minutes per day	Gestation 8-16 weeks (~28 weeks, to term)	Standard text messages	Physical activity specific texts	Women were able to keep the Fitbit as compensation for participating	5.9%*	NA	PP	Ineffective – no differences in physical activity
Olsen et al 2018	Integrative Model of Behavior	To prevent excessive GWG	Diet and physical activity	1)GWG – difference between first	Gestation<20 weeks	Website with health information,	Control website plus weight gain tracker, diet and	Participants received financial	2.5%	46.1% logged in once every	ITT & PP	Ineffective – no differences in total GWG

	Prediction and the Behavior Model for Persuasive Design			weight at <14 weeks and last weight at >37 weeks - from medical records and compliance with IOM guidelines	(~28 weeks, to term)	local resources, blogging tool, appointment reminder	physical activity goal setting, self-monitoring, blogging tool	incentives for enrolling in the study and for each online questionnaire they completed – up to $45		45 days; median log in rate was 5.6% of days= 10 times; median page views 6 for BCT pages; 27% consistent users of weight monitoring feature		or % exceeding GWG guidelines
Dahl et al 2018	Unified Theory of Acceptance and Use of Technology	To encourage adequate GWG	12 weekly diet and physical activity challenges	1)GWG - difference between pre-pregnancy and final weight before delivery, self-reported with photo of scales verification	Gestation<20 weeks (12 weeks)	Intervention targeting stress reduction and management	12 weekly behaviour change challenges via Make-me app; virtual teams; website with weight gain	None	38.9%	NA	PP	Ineffective – non-significant higher % within GWG guidelines and significant positive effect on healthy eating

				2)Rapid eating and Activity Assessment for Participants Short Scale 3)Godin Leisure-Time Exercise Questionnaire			tracker and information					behaviours or physical activity for intervention group

* Intervention and control combined
