# Supplementary material for: Exclusively Digital Health Interventions Targeting Diet, Physical Activity, and Weight Gain in Pregnant Women: Systematic Review and Meta-Analysis
Source: JMIR Mhealth Uhealth. 2020 Jul 10;8(7):e18255. doi: 10.2196/18255 (PMC7382015; doi:10.2196/18255)

**Multimedia Appendix 4: Subgroup analyses.**

**Figure MA 4.1 Studies where no BCTs were apparent in onboarding session**
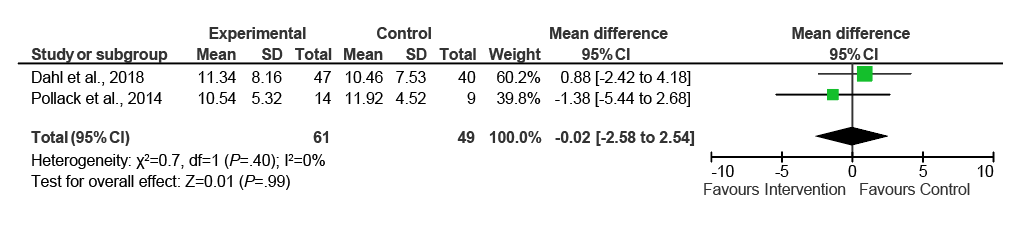


**Figure MA 4.2 Studies where BCTs were apparent in onboarding session**


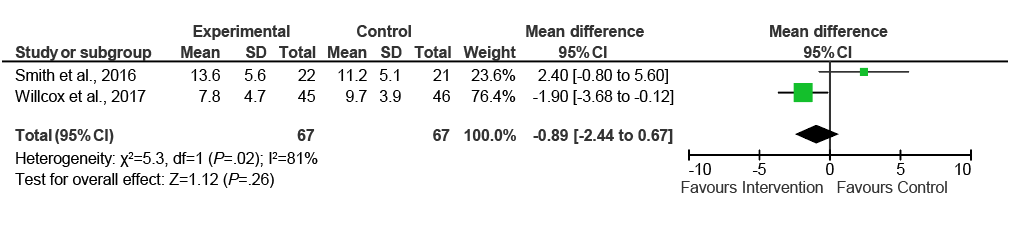

Supplement: Multimedia Appendix 4 [file mhealth_v8i7e18255_app4.docx]
