# Supplementary material for: Exclusively Digital Health Interventions Targeting Diet, Physical Activity, and Weight Gain in Pregnant Women: Systematic Review and Meta-Analysis
Source: JMIR Mhealth Uhealth. 2020 Jul 10;8(7):e18255. doi: 10.2196/18255 (PMC7382015; doi:10.2196/18255)
Supplement: Multimedia Appendix 5 [file mhealth_v8i7e18255_app5.pdf]

Multimedia Appendix 5: Summary of BCTs.

**BCT**	Effective			Non effective								
	Willcox et al 2017	Redman et al 2017	Hayman et al 2017	Evans et al 2012	Pollack et al 2014	Evans et al 2015	Smith et al 2016	Choi et al 2016	Huberty et al 2017	Olson et al 2018	Dahl et al 2018	Total
1.1 Goal setting (behaviour)	x	x	x		x		x	x		x	x	8
1.2 Problem solving	x	x	x				x	x		x		7
1.3 Goal setting (outcome)	x	x			x					x	x	5
1.4 Action planning	x		x		x					x	x	5
1.5 Review behaviour goal(s)	x	x	x									3
1.6 Discrepancy between current behaviour & goal		x										3
1.7 Review outcome goal(s)		x										1
**Goals and Planning total**	**5**	**6**	**4**	**0**	**3**	**0**	**2**	**2**	**0**	**4**	**3**	**29**
2.2 Feedback on behaviour	x	x	x		x			x		x		6
2.3 Self-monitoring of behaviour	x	x			x		x	x		x	x	7
2.4 Self-monitoring of outcome(s)	x	x			x			x		x	x	7
2.7 Feedback on outcomes		x								x		2
**Feedback and Monitoring total**	**3**	**4**	**1**	**0**	**3**	**0**	**1**	**3**	**0**	**4**	**2**	**21**
3.1 Social support	x	x	x					x	x		x	6
4.1 Instructions on how to perform a behaviour	x	x					x	x	x	x	x	7
4.2 Information about antecedents		x	x									2
5.1 Information about health consequences	x	x	x	x	x	x	x	x	x	x	x	11
5.6 Information about emotional consequences	x	x	x				x		x		x	6
6.1 Demonstration of the behaviour								x				2
6.2 Social comparison	x										x	2
7.1 Prompts/cues	x							x				2
8.2 Behaviour substitution									x	x	x	3
10.9 Self-reward	x									x	x	3
12.1 Restructuring physical environment	x		x									2
15.3 Focus on past success	x											1
**Total number of BCTs**	**17**	**15**	**10**	**1**	**7**	**1**	**6**	**10**	**5**	**12**	**12**	
